# Current trends in the etiology and diagnosis of HPV-related head and neck cancers

**DOI:** 10.1002/cam4.424

**Published:** 2015-02-01

**Authors:** Ryan C Chai, Duncan Lambie, Mukesh Verma, Chamindie Punyadeera

**Affiliations:** 1 The University of Queensland Diamantina Institute, The University of Queensland, The Translational Research InstituteWoolloongabba, Queensland, 4102, Australia; 2 IQ PathologyWest End, Queensland, 4101, Australia; 3 Division of Cancer Control and Population Sciences, National Cancer Institute (NCI), National Institutes of Health (NIH)9609 Medical Center Drive, Rockville, Maryland, 20850; 4 Institute of Health and Biomedical Sciences, Queensland University of TechnologyVictoria Park Rd, Kelvin Grove, Queensland, 4059, Australia

**Keywords:** Biomarkers, epidemiology, HPV, oropharyngeal cancer, saliva diagnostics

## Abstract

Human papilloma virus (HPV) infection is a major risk factor for a distinct subset of head and neck squamous cell carcinoma (HNSCC). The current review summarizes the epidemiology of HNSCC and the disease burden, the infectious cycle of HPV, the roles of viral oncoproteins, E6 and E7, and the downstream cellular events that lead to malignant transformation. Current techniques for the clinical diagnosis of HPV-associated HNSCC will also be discussed, that is, the detection of HPV DNA, RNA, and the HPV surrogate marker, p16 in tumor tissues, as well as HPV-specific antibodies in serum. Such methods do not allow for the early detection of HPV-associated HNSCC and most cases are at an advanced stage upon diagnosis. Novel noninvasive approaches using oral fluid, a clinically relevant biological fluid, allow for the detection of HPV and cellular alterations in infected cells, which may aid in the early detection and HPV-typing of HNSCC tumors. Noninvasive diagnostic methods will enable early detection and intervention, leading to a significant reduction in mortality and morbidity associated with HNSCC.

## Introduction

Head and neck squamous cell carcinoma (HNSCC) includes malignancies in five major anatomic sites, namely, oral cavity, oropharynx, nasopharynx, hypopharynx, and larynx. HNSCC is the sixth most common malignancy with an estimated annual incidence of ∽633,000 and 355,000 deaths worldwide 1. HNSCCs are highly curable if detected early and the most common treatments include surgery, radiation therapy, chemotherapy, or combinations of these three treatments. In the initial stages of the disease, a patient may not show any clinical symptoms and as such a significant number of patients present with metastatic disease at the time of diagnosis (regional nodal involvement in 43% and distant metastasis in 10%), leading to 5-year survival rates of less than 60% 2.

Oral squamous cell carcinoma (OSCC) and oropharyngeal squamous cell carcinoma (OPSCC) are the most common types of HNSCC, accounting for 263,900 new cases and 128,000 deaths worldwide in 2008 3. Tobacco smoking is a major risk factor for HNSCC with ∽80% of cases attributed to tobacco exposure 4. Alcohol consumption is also a risk factor for HNSCC 5, which can act synergistically with tobacco to increase the risk of HNSCC 6. In recent decades, the overall incidence of HNSCC is in the decline in the developed world due to a reduction in the consumption of tobacco. However, there is a concomitant increase in the incidence of OPSCC as a result of human papilloma virus (HPV) infection. Unlike tobacco-related HNSCC, patients with HPV-associated OPSCC are usually less likely to have any history of excess tobacco or alcohol consumption. Instead, about 60% of OPSCC patients in the western world are positive for the oncogenic forms of HPV, in particular the 16 subtypes 7. It is estimated that tumors in the oropharynx are five times more likely to be HPV-positive than those in the oral cavity, larynx, or hypopharynx 8. HPV-positive HNSCCs have genetic alterations that are direct result of HPV oncoproteins, E6 and E7, which inactivate the tumor suppressor gene products, p53 and Rb, respectively. In addition, HPV-positive HNSCCs also vary in their allelic and chromosomal stability as well as global gene expression profiles and DNA methylation profiles 9. Patients with HPV-positive OPSCC have a better prognosis and response to therapy compared to HPV-negative patients. Loco-regional control is also significantly better in HPV-positive OPSCC, but the rate of distant metastasis increases after 2 years unlike in patients with HPV-negative tumor 10. Furthermore, metastases are more likely to occur significantly later in HPV-positive OPSCC compared to HPV-negative tumors, involving multiple organs, such as the skin, intra-abdominal lymph nodes, and brain 10. However, the implication of HPV status in nonoropharyngeal sites on prognosis and distant metastasis remains unclear. Even though HPV has now been recognized as an independent risk factor for a subset of HNSCC, the HPV-related oncogenic pathways that influence HNSCC biology are still not well understood. Treatment strategies in the future may target specific molecular pathways that differ between HPV and non-HPV-associated HNSCC, which highlights the importance of the accurate identification of this distinction between the two different HNSCC subtypes. This review summarizes the current knowledge on the oncogenic pathways associated with HPV and how these insights have translated into the current regime of diagnostics for HPV-positive HNSCC. Furthermore, novel approaches involving the use of oral fluid and new molecular biomarkers may pave the way for better screening and diagnostic strategies for HPV-positive HNSCC patients.

## Epidemiology of HPV-Positive OPSCC

The significant increase in HPV-associated OPSCC over the last decade among nonsmoking, young individuals 11 reflects an increasing prevalence of oral HPV infection as a causative factor, possibly due to changes in sexual behavior 12,13. Studies have shown a varying prevalence of HPV infection in various population groups living in different geographical regions, that is, 0.6% in Japan (4/662 individuals of various ages from Miyako Island) 14, 6.9% in the United States (385/5579 individuals aged 14–69) 15, 9.3% in Sweden (45/483 individuals aged 15–23) 16, and 2.3% in Australia (7/307 individuals aged 18–35) 13.

Most HPV infections do not progress to cancer, however, delayed clearance of infection has been shown to be a risk factor for the development of anogenital cancer 17,18. The incidence of HPV-positive OPSCC is increasing significantly, with an estimated 22,000 cases with HPV positivity from 85,000 OPSCC cases worldwide in 2008 19. Between 1988 and 2004, there was a 225% increase in the incidence of HPV-positive OPSCC, and a simultaneous 50% decrease in HPV-negative OPSCC in the United States 11 with similar trends recorded in Europe and Australia 20,21. It has been estimated that 25.6% of OPSCC worldwide are HPV-related and this may vary by geographical regions 22. The proportion of HPV-positive OPSCC was 56% in North America; 52% in Japan; 45% in Australia; 39% in Northern and Western Europe; 38% in Eastern Europe; 17% in Southern Europe; and 13% in the rest of the world 22.

The incidence of HPV-positive OPSCC is the highest among middle-aged, nonsmoking white males (40–59 years old) with higher socioeconomic status and multiple sexual partners 23. Patients with HPV-positive OPSCC are less likely to consume tobacco and alcohol compared to patients with HPV-negative OPSCC 12,24. HPV-positive OPSCC patients have on average more than 8–10 sexual partners with a history of more than four oral sexual partners 25–28. Sexually acquired HPV is generally cleared on its own but in few cases viral DNA is integrated into the host genome, which is a key step in HPV-induced carcinogenesis.

HPV positivity in OPSCC is associated with a reduction in the risk of death compared to patients with HPV-negative tumors. A study has shown that patients with HPV-positive OPSCC had a higher 5-year disease-free survival (75% vs. 14%) and overall survival (79% vs. 19%) compared to patients with HPV-negative tumors 29. The more favorable outcome for patients with HPV-positive OPSCC is associated with improved response to chemotherapy (82% vs. 55%) and chemoradiation (84% vs. 57%) compared to HPV-negative patients 30. The prognostic advantage of HPV-positive OPSCC is partly attributed to the patient population affected—which is the younger age group, decreased tobacco consumption, and a higher performance status. However, in studies adjusted for these confounders, the improved prognosis of HPV-positive OPSCC still persists 10, indicating a fundamental biological difference between HPV-positive and -negative diseases, which remains unclear.

HPV L1, the major capsid protein that can self-assemble into virus-like particles (VLP) is the basis for HPV vaccines that generate neutralizing antibodies against the VLP proteins 31. There has been strong evidence from clinical trials supporting this argument for both the bivalent HPV16/18 vaccine and quadrivalent HPV 6/11/16/18 vaccine against cervical, vaginal, and vulvar infections in women 32,33. The quadrivalent vaccine has also been shown to be effective against anogenital HPV infections in men 34,35. Due to the fact that oropharyngeal HPV infection is largely associated with sexual behaviors, the decrease in prevalence of genital HPV infection by the vaccine might indirectly reduce the incidence of oral HPV infection, independent of the potential direct effect of the vaccine on oropharyngeal HPV infection. To date, there is only one study that has assessed the effect of HPV vaccine against oropharyngeal HPV infections 36. Among patients who participated in this trial, vaccine efficacy (VE) against oral HPV16/18 infection was 93% (1/2910 infection in vaccinated group and 15/2924 in the control group) 36. This study provided proof-of-principle that HPV vaccine may prevent HPV-induced oral and oropharyngeal cancers. However, the current population age group of >30 years may not benefit from HPV vaccination because prophylactic vaccines do not clear HPV infections.

## HPV Biology and Malignant Transformation by E6 and E7 Viral Oncoproteins

HPV is a member of the Papillomaviridae family, small DNA viruses that are commonly detected in various species including birds and mammals 37. HPVs exclusively infect human epithelial cells and are associated with different anatomic site preferences for either cutaneous or mucosal squamous epithelium 38. High-risk (HR) HPV types, such as HPV-16, -18, -31, and -33, are commonly found in cervical squamous cell carcinomas 39, other anogenital malignancies 40, and in a subset of HNSCC 39.

The HPV genome consists of a small double-stranded DNA of ∽8000 base pairs, divided into three major regions. The early genes (E1–E7) are expressed early in the viral infectious cycle for the regulation of transcription, plasmid replication, and transformation. The late genes code for the major (L1) and minor (L2) capsid proteins, involved in the packaging of viral genome and virus release. The long control region (LCR) contains the regulatory elements for transcription and replication. E6 and E7 encode the main HPV oncoproteins that promote cell cycle progression and viral DNA replication. As most people with HPV infection do not develop cancers, expression of E6 and E7 is necessary but not sufficient for malignant transformation. However, increased proliferative capacity and evasion of apoptosis induced by E6 and E7 can lead to the accumulation of DNA damage and mutations that can ultimately result in malignant transformation and carcinogenesis.

The major role of high-risk E7 protein is to reprogram terminally differentiated epithelial cells at the surface epithelium in order for the host cells to re-enter the cell cycle, which is required for viral DNA replication. E7 binds to the retinoblastoma (Rb, a tumor suppressor protein) and other members of the Rb family, such as p107 and p130. Proteins from the Rb family regulate the G1-S phase transition through interaction with the E2F family of transcription factors, which in turn control many genes that are involved in regulating cell cycle progression, differentiation, mitosis, and apoptosis 41. The binding of E7 disrupts the Rb-E2F complex, leading to the inactivation of Rb through proteasomal degradation. The release of E2F also subsequently activates responsive genes, such as cyclin A and cyclin E, which promotes the entry of cells into S phase 42,43. E7 protein also leads to increased induction of p16^INK4a^, which is routinely used as a biomarker for HPV-associated lesions and cancers. p16^INK4a^ is a tumor suppressor in normal cells, but it has been shown to be essential for the survival of HPV-positive cervical cancer cell lines 44. This oncogenic effect of p16 depends on the inhibition of CDK4/CDK6 in cancer cells where Rb is inactivated, suggesting the presence of CDK4/6 substrates that may cause cell death when phosphorylated in cells with inactivated Rb 44.

Aberrant cell proliferation and DNA synthesis in the absence of sufficient growth signals as observed in differentiating HPV-infected cells can trigger p53-dependent apoptotic programs. The inactivation of Rb by E7 protein sensitizes cells to p53-dependent apoptosis, but E6 protein targets p53 for degradation, thus inhibiting the proapoptotic functions of p53 45. E6 binds directly to E6-associated protein (E6-AP), a specific ubiquitin-ligase for p53 degradation. A study has shown that the repression of HR E6 and E7 expression in oropharyngeal cancer cells is associated with restoration of p53 and Rb tumor suppressor pathways and increased apoptosis 46. In addition, E6 and E7 also interfere with growth inhibitory cytokines released by host cells upon HPV infection, such as tumor-necrosis factor-*α* (TNF*α*). TNF*α* activates the extrinsic apoptotic pathway through TNF receptor 1 (TNFR1), Fas cell surface death receptor (FAS) and the TNF-related apoptosis-inducing ligand (TRAIL) receptors. E6 abrogates the apoptotic effect of TNF*α* by binding to TNFR1, which inhibits the subsequent transduction of apoptotic signals 47. E6 can also disrupt the mitochondrial apoptotic pathway by interactions with the proapoptotic Bcl2 members BAK and BAX as well as inducing the expression of inhibitors of apoptosis proteins (IAPs) and survivin 48.

The expression of E6 and E7 can result in the immortalization of host cells but it is insufficient to directly transform cells. HR E6 and E7 independently induce genomic instability in normal cells 49, which is a necessary step for malignant transformation. The expression of E6 and E7 has been shown to result in mitotic defects, such as multipolar mitoses, anaphase bridges, and aneuploidy 50. Under normal circumstances, cells with mitotic defects are targeted for cell death. Through the actions of E6 and E7 on cell cycle checkpoints and apoptosis, cells with abnormal centrosomes are allowed to survive and accumulate 51,52. E6 and E7 can also induce DNA damage and increase the frequency of foreign DNA integration into the host genome 53,54. The activation of ATM–ATR pathway (ataxia telangiectasia-mutated—ATM and RAD3-related DNA damage repair pathway)-dependent DNA damage response is important for the replication of differentiation-dependent viral genome, but not the stable maintenance of episomes in undifferentiated epithelial cells 55. Furthermore, E7 can abrogate ATM–ATR-induced cell cycle checkpoints to promote cell cycle progression regardless of the presence of DNA damage, leading to genomic instability and malignant progression 56.

## Current Diagnostics for HPV-Positive HNSCCs

Currently, there is no consensus on the optimal way to identify HPV-positive HNSCC. Different methods include the detection of p16 protein expression using immunohistochemistry (IHC) as well as HPV-related genetic material using polymerase chain reaction (PCR) and in situ hybridization (ISH) in tumor biopsy samples. In addition, the presence of HPV-specific antibodies in serum has also been associated with increased risk of developing OPSCC 28,57.

### p16 immunohistochemistry

During immortalization of host cells, the E7 protein of HR-HPV binds to Rb, resulting in the compensatory overexpression of the tumor suppressor gene p16 in HPV-infected tumor cells 58. The IHC analysis of p16^INK4A^ in HNSCC tumor biopsies has been shown to serve as a surrogate marker to identify HPV infection in histologic preparations from HNSCCs 59. However, in a pooled analysis of 496 patients with OPSCC from different studies utilizing DNA-based HPV testing, 5% of cases were p16^INK4A^-positive/HPV-negative and 8% were p16^INK4A^-negative/HPV-positive 60. Another study has shown that p16^INK4A^ is also overexpressed in a subset of HNSCC lacking HPV DNA, with close to 14% of tumors that were p16-positive were negative by HPV-specific ISH and PCR 61. Strikingly, Hoffmann et al. reported that no overexpression of p16^INK4A^ was observed in 3/14 (21.4%) patients who were positive for HPV DNA and mRNA 62. Furthermore, Harris et al. demonstrated an overexpression of p16 in young patients with oral tongue SCC without evidence of HPV infections 63. Liang et al. also showed that OPSCC patients who were p16-positive and seronegative for HPV antibodies had significantly increased hazard of all causes of death 64. These data support the notion that p16 overexpression alone is not sufficient to accurately identify HPV infection in HNSCC. However, p16 IHC has been shown to be a suitable test for risk stratifying patients with OPSCC as p16 positivity in tumor correlates with better survival 65. p16 IHC has been adopted as a single test of choice for many medical practitioners due to the fact that it has been extensively studied and cost effective with clear staining interpretation guidelines 65. Direct identification of HPV using DNA and RNA-based methods will still be required for clinically relevant infection and may replace p16 IHC or used in conjunction with it in the future 65.

### HPV DNA detection

PCR is a highly sensitive and cost-effective method for the detection of HPV. Due to the high number of HPV strains, primers targeting the conserved L1 open reading frame are commonly used to detect a broad spectrum of HPV, such as the MY09/MY11 and GP5/GP6 primer pairs 66,67. The MY09/MY11 primer pair is synthesized with several degenerate nucleotides in each primer and is thus a mixture of 25 primers capable of amplifying a wide spectrum of HPV types 68. In contrast, there are only two primers, GP5/GP6 and the detection of multiple HPV strains is achieved by lowering annealing temperature during PCR 68. Due to the use of consensus sequences of L1 from multiple subtypes, specific genotyping is not possible with MY09/MY11 and GP5/GP6 primer pairs. Moreover, the L1 region of the HPV genome may be deleted upon viral integration into the host genome, hence decreasing the sensitivity of HPV detection. PCR strategy targeting the tumorigenic E6 and/or E7 sequence, which is retained by infected cells through viral genome integration, may prove to be more sensitive in the detection of HPV. This was highlighted by a study of HPV detection in anal carcinoma that showed a lower detection rate with L1 consensus primers (16%), whereas strain-specific E6 primers yielded a HPV positivity of 46% 69.

E6 and E7 regions have been shown to harbor many sequence variations between HPV types 70. Suitable primers directed toward E6/E7 of different HPV strains can be designed to not only determine if HPV is present but also—at the same time—to distinguish between HR and low-risk strains. However, standard PCR techniques have low specificity and do not allow for a distinction between tumor-derived or healthy stroma-derived HPV. Furthermore, PCR techniques are not able to distinguish between episomal and integrated HPV DNA, thus decreasing the ability to detect clinically relevant infection. Currently, there are no standardized PCR-based methods in clinical application, leading to varied analytical sensitivities and specificities of PCR-based assays between laboratories. However, studies have shown that when used in conjunction with a standardized protocol and quality-controlled reagents, PCR-based HPV detection methods demonstrated good interlaboratory agreement 71.

Another method of HPV DNA detection in tumor samples is ISH. The advantage of ISH over a PCR method is the high specificity due to the reliable detection and identification of HPV in topographical relationship with their pathological lesions 72. The result of ISH can be evaluated microscopically and the appearance of precipitate within the nuclei of epithelial cells is indicative of HPV presence 72. Furthermore, integrated and episomal HPV DNA can be distinguished by the presence of punctate or diffuse signals, respectively 72. As discussed in the previous section, HPV DNA presence as detected by ISH was significantly correlated with p16 IHC 73. In spite of the high specificity of this method (100%), the sensitivity is low (86%) with an estimated 13–41% false-negative rate in HNSCC 74,75.

### HPV RNA detection

The expression of mRNA from integrated and episomal HPV DNA indicates that viral oncogenic transcripts are crucial in tumor initiation and progression 76. Therefore, PCR methods targeting HPV mRNA is a better approach than DNA-based methods in providing evidence of clinically relevant HPV infection. A study by Deng et al. showed that E6/E7 transcripts were detected only in 15/54 (27.5%) of the HPV-positive tumor samples, which also correlated with high HPV16 DNA load 77. Another study by Holzinger et al. showed that E6/E7 transcripts were detected in 48/96 (50%) OPSCC tumor samples tested positive for HPV DNA 78. Consistent with the findings of Deng et al., Holzinger et al. showed a significant positive correlation between high viral load and E6/E7 mRNA expression levels. Moreover, E6/E7 mRNA expression has been shown to be highly expressed in HPV-infected tonsillar SCC (75%) and lower expression was observed in other oropharyngeal areas 77. This might indicate that E6/E7 mRNA expression is more prevalent in tonsillar carcinomas. Due to the instability of RNA and the suboptimal preservation of routine biopsy samples using formalin-fixed paraffin-embedded (FFPE) analyses, the methods discussed thus far for HPV transcript detection rely on the analysis of fresh-frozen tissue in research laboratories, therefore hampering the translation to routine clinical diagnostic. More recently, HR HPV E6/E7 mRNA ISH has been developed as a potential detection tool in FFPE tissues. In a study of 196 OPSCC patients, RNA ISH was positive in 147/148 (99.3%) of the p16-positive tumors, and demonstrated a better sensitivity in HPV detection than DNA ISH 79. Concordantly, Schache et al. reported sensitivity and specificity of 97% and 93%, respectively, in the detection of HR-HPV using RNA ISH in FFPE OPSCC samples 80. These data demonstrated that the detection of HPV transcripts is highly concordant with active and clinically relevant HPV infection, which can be incorporated into current clinical diagnostics.

The specificity of HPV-specific DNA and RNA ISH for HPV infection in tumor samples in combination with the high sensitivity of p16 IHC or HPV DNA PCR can be an effective diagnostic strategy for HPV-associated HNSCC. It has been suggested that screening tumors with p16 IHC to be performed first, and upon positive result, to be followed by HPV-specific test, such as ISH or PCR 73,81. This will provide evidence that HPV is in the tumor and that the HPV is transcriptionally active based on p16 overexpression and E6/E7 mRNA expression levels 65.

### HPV serology

The detection of HPV-specific IgG in serum is a useful biomarker to determine previous and current HPV infection status 82. Serological biomarkers are not site-specific, and can arise due to HPV infections at sites other than the oral cavity, hence potentially affecting the specificity of the assay. However, an earlier study involving 900,000 subjects has shown a significant association between the presence of oncogenic HPV-specific antibodies and an increased risk of HNSCC 57. An earlier study by Cameron et al. showed that the presence of HPV-specific immunoglobulin G (IgG) in serum and saliva was observed in HIV-positive individuals with elevated risk for HPV infections 83. A more recent study has shown a strong correlation between HPV16 E1, E2, and E7 antibody levels in OPSCC patients compared to healthy controls 84. Furthermore, OPSCC patients with HPV16 E6 and E7 seropositivity also showed a more favorable all-cause survival compared to seronegative patients 64. Collectively, seropositivity of HPV-specific antibodies is a potential surrogate marker and prognostic marker for HPV-associated OPSCC.

### HPV detection in oral fluid

Most of the current diagnostic assays are designed for excised tumor tissues obtained at the time of surgical biopsy or resection. More recently, the use of biological markers in oral fluids for the detection of HPV-associated HNSCC has been gathering a lot of attention due to the noninvasive and cost-effective nature, as well as the proximity to oral tumors, which allows for early cancer detection and monitoring of disease progression 85. Oral fluid has been shown to contain different analytes, such as hormones, steroids, antibodies, growth factors, cytokines, chemokines, and drugs, that may reflect local and systemic disease states 86–88. It also contains whole cells, genetic materials, as well as proteins that may allow for the detection of HPV and cellular alterations in infected cells, which may aid in early detection and HPV-typing of HNSCC tumors. Recent studies on the detection of HPV in oral fluid for the diagnosis of HNSCC are outlined and summarized in Table1.

**Table 1:** Recent studies on the detection of HPV in oral fluid of HNSCC patients

Study	Tumor HPV DNA	Saliva HPV DNA	Detection method	Summary of findings
Smith et al. 23,89	38/190 (20%)	57/190 (28.4%)	PCR of L1	HR-HPV in oral rinse is a risk factor for HNSCC independent of alcohol and tobacco consumption
Zhao et al. 90	28/92 (30.4%)	16/92 (17.4%)	RT-qPCR of HPV16 E6 and E7	Quantitative analysis of HR-HPV DNA allows for the detection of HPV-associated HNSCC, but is not predictive of HNSCC in general
Chuang et al. 91	20/59 (33.9%) pretreatment	2/20 (10%) posttreatment	RT-qPCR of HPV16 E6 and E7	Patients with HR-HPV DNA in oral rinse posttreatment are at significant risk for tumor recurrence. Small sample size
Agrawal et al. 92	44/135 (32.6%) pretreatment	30/133 (22.6%) pretreatment, 37/135 (27.4%) posttreatment	PCR of L1 and hybridization to a linear probe array	HR-HPV DNA were more likely to be present in oral rinse of HPV+ than HPV− HNSCC patients before and after therapy but is not a prognostic marker for recurrence
Adamopoulou et al. 96	Not performed	7/68 (10.3%) in oral cancer, 12/34 (35.3%) in HIV+	PCR of L1 and genotyping by RFLP	The detection rate of HPV in saliva is higher in HIV+ individuals than patients with oral cancer
Tachezy et al. 94	53/86 (61.6%)	37/86 (43%)	PCR of L1 and genotyping reverse line blot hybridization (RLB)	The presence of HPV DNA in oral rinse is significantly correlated with the presence of HPV DNA in tumor and HPV-specific antibodies in sera
Fakhry et al. 95	Not performed	48/91 (52.7%) precancerous lesions, 72/1524 (4.7%) from multiple follow-ups in 401 HIV+ individuals	PCR of L1 and HPV16 E6	The combination of HPV16 and abnormal cytology detected in oral samples (tonsillar brush and oral rinse) of patients with precursor lesions, but not HIV+ individuals, was associated with OSCCs
Koslabova et al. 93	83/141 (58.9%)	64/83 (77.1%) pretreatment, 63/83 (75.9%) posttreatment	PCR of L1 and sequencing	HPV DNA presence in oral rinse and the presence of HPV-specific antibodies correlate with HPV infection in tumor tissues, but HPV presence cleared up in oral rinse 1 year posttreatment. Sustained seropositivity for HPV16 oncoproteins posttreatment is a more specific marker for recurrence
Nordfors et al. 97	22/29 (75.9%) tonsillar carcinoma, 16/18 (88.9%) base of tongue carcinoma	18/29 (62.1%) tonsillar carcinoma, 8/18 (44.4%) base of tongue carcinoma	PCR of L1 and HPV16 E6; Bead-based multiplex assay for HPV genotyping	The presence of HPV DNA in oral samples is significantly correlated with HPV-positive tumors in the tonsillar and base of tongue region

HR-HPV, high-risk human papilloma virus; RT-qPCR, real-time quantitative polymerase chain reaction; HNSCC, head and neck squamous cell carcinoma.

An earlier study of oral exfoliated cells and tumor tissues from HNSCC patients (*n* = 201) revealed that there was a significant correlation between HR-HPV detected in oral rinse and the HR-HPV types present in tumor tissues 89. The data suggest that an assessment of HPV genotypes in oral rinse maybe predictive of HPV-associated HNSCC, and have shown that HPV infection is a risk factor for HNSCC independent of alcohol and tobacco use. Compared to the use of swabs or scrapes to collect mucosal cells from a limited number of oral sites, the use of oral rinse is likely to have sampled the tumor site and the localized field of HPV infection 89. Another study utilized real-time quantitative PCR (RT-qPCR) to detect HPV16 E6 and E7 DNA in oral rinses as a screening method for HNSCC 90. RT-qPCR enables a more accurate quantification of HPV DNA copy number present in samples and hence an improved sensitivity for HPV detection compared to nonquantitative amplification methods. A total of 42/92 (45.7%) primary tumor tissues and 30/92 (32.6%) oral rinses from HNSCC patients had detectable HPV16 DNA.

HPV DNA detection in tumor tissue and oral rinse from patients with tumors demonstrated a significant correlation (*P* < 0.001). However, the authors noted that some tumors that were HPV-positive did not yield HPV positivity in the oral rinses, and that the oral rinse level of HPV16 was significantly lower than that of the tumor, presumably due to the diluting effect of normal exfoliated cells unrelated to the tumor cells 90.

Chuang et al. examined HNSCC tumors and paired pre- and posttreatment oral rinse samples from 59 patients for HPV16 using RT-qPCR for the diagnosis of persistent and recurrent HNSCC 91. Before treatment, 20/59 (33.9%) patients were HPV16-positive in their tumors. After treatment, 4/20 (20%) of these patients ultimately developed recurrence and 2/4 (50%) were HPV16-positive in surveillance oral rinses, with an assay sensitivity and specificity for recurrence based on HPV positivity of 50% and 100%, respectively, albeit with a small sample size. Another study has found no association between oral HPV infection after therapy and tumor recurrence, due to the fact that the majority of the HPV infection detected in oral rinse after therapy was not identical to that found in tumor 92. However, a recent prospective study demonstrated that HPV detection in oral rinse was comparable to that of HPV presence in tumor tissues 93. In addition, the presence of antibodies specific to viral oncoproteins in sera was found to be correlated with favorable prognosis and lower frequency of recurrence 93. This was consistent with an earlier study that showed the presence of HPV DNA in tumor tissues and in oral rinse samples and was associated with the presence of HPV-specific antibodies in sera 94. Despite the low assay sensitivity for tumor recurrence due to the low sampling size of the studies, these data have exhibited the feasibility of correlating HR HPV DNA positivity in oral rinses and HPV-related antibodies in blood for the detection and surveillance of disease progression.

A study by Fakhry et al. aimed to develop a cytology test for the detection of HPV-associated HNSCC from populations with elevated risk of developing OPSCC, namely patients with abnormal oropharyngeal lesions and HIV-infected patients 95. Using a PCR-based method with consensus MY09/MY11 and HPV16-specific primers, the authors have found that HPV16 was present in tonsillar brushings and oral rinses in 48/91 (52.7%) patients with abnormal oropharyngeal lesions and in 72/1524 (4.7%) specimens from HIV-infected patients (*n* = 401) without such lesions. Another study also included the HR group of HIV-positive patients and found that HPV DNA was detected in saliva of 7/68 (10.3%) of oral cancer patients and in 12/34 (35.3%) HIV-positive individuals 96. The presence of HR-HPV DNA in oral samples may therefore be a strong biomarker for the development of OPSCC in HR groups with premalignant lesions and immuno-compromised individuals.

A recent study analyzed and correlated the presence of HPV DNA in oral samples (oral rinses and/or tonsillar swabs) in patients with the incidence of HPV-positive tonsillar and base of tongue cancer 97. The presence of HPV DNA in oral samples that is concordant with HPV positivity in tumor samples was detected in 18/22 (76%) and 8/16 (50%) of patients with tonsillar and base of tongue cancer, respectively. This is consistent with the fact that the majority of HPV-positive HNSCC is found in the tonsillar and base of tongue regions 98,99, demonstrating the site specificity of HPV detection in oral rinse.

Studies to date have collectively demonstrated promising data for the utilization of oral fluid as a valid specimen for the detection of HR HPV as well as other prognostic markers. Currently, there is only one laboratory-based salivary diagnostic test available to detect HPV (OralDNA Labs, Eden Prairie, MN, USA) that determines the risk of developing HPV infection by detecting different strains of HPV such as HPV8, 11, 16, and 18 via PCR using oral rinse samples. However, the clinical performance interims of the sensitivity and specificity of this test and other oral fluid-based detection methods lack experimental evidence. It is also important to experimentally validate if HPV infection is current/active or in the past. Oral fluid based tests to determine HPV infection require further improvements due to the different origins of the cells being tested in oral fluid, namely HPV-positive tumor cells, any associated HR-HPV infection that can lead to the development of oral cancers or an independent HR HPV infection, as well as the percentage of host healthy exfoliated cells and immune cells 72.

## Conclusions

HPV-associated HNSCC represents a distinct entity from tobacco and alcohol-related HNSCC. The expression of HR-HPV oncoproteins E6 and E7 upon viral infection and integration results in the immortalization of host cells and subsequent malignant transformation through increased proliferative capacity, upregulation of antiapoptotic pathways and increased genomic instability. Currently, the treatment plans for nonoropharyngeal HNSCC patients do not change with respect to HPV status. However, the improved outcome in patients with HPV-positive OPSCC has been shown in many studies and the de-escalation of treatment for this subset of HNSCC has been proposed 100, further highlighting the importance of HPV testing. The presence of HR-HPV DNA and HPV-associated protein markers in tumor biopsy are currently being utilized to diagnose HPV-positive HNSCC. Oral fluid presents a promising noninvasive alternative for the detection of oncogenic HPV DNA that correlates significantly with HPV infection in tumors in recent studies. However, further development and validation of HPV detection in oral fluid is warranted due to the low sensitivity and specificity for clinically relevant HPV infection before being implemented as a clinical diagnostic medium. The incorporation of novel screening methods into the current diagnostic regime will enable the early detection and intervention, risk assessment, and response to treatment of HNSCC.
